# Outcomes of Two Different Negative Pressure Therapy Systems for Closed Incision Management in Knee and Hip Arthroplasty: A Systematic Review and Meta-Analysis

**DOI:** 10.7759/cureus.40691

**Published:** 2023-06-20

**Authors:** H. John Cooper, Leah P Griffin, Christine Bongards, Ronald Silverman

**Affiliations:** 1 Orthopedics, Columbia University Irving Medical Center, New York, USA; 2 Medical Solutions Division, 3M, San Antonio, USA; 3 Health Economics and Reimbursement, 3M, Neuss, DEU

**Keywords:** lower ssi rate, wound healing, total knee arthroplasty, total joint arthroplasty, total hip arthroplasty, surgical site infection, surgical site complication, foam dressing, cinpt

## Abstract

Closed incision negative pressure therapy (ciNPT) has been adopted into practices of diverse surgical specialties to help reduce postsurgical complication risks. There are two primary commercially available systems that deliver ciNPT through different mechanisms. The purpose of this meta-analysis is to compare the potential effects of two different ciNPT systems on clinical outcomes following hip and knee arthroplasty. A systematic literature search was conducted to identify hip and knee arthroplasty studies comparing the incidence of surgical site infections (SSIs) and surgical site complications (SSCs) versus standard of care (SOC) following the use of two different ciNPT systems. Four meta-analyses were performed by calculating risk ratios (RR) to assess the effect of (1) ciNPT with foam dressing (ciNPT-F) versus SOC and (2) ciNPT with multilayer absorbent dressing (ciNPT-MLA) versus SOC. Comprehensive Meta-Analysis Version 3.0 (Biostat Inc., Englewood, NJ) software was used to perform the analyses. Twelve studies comparing ciNPT-F to SOC and six studies comparing ciNPT-MLAto SOC were analyzed. SSI rates were reported in seven of 12 studies involving ciNPT-F. In those, ciNPT-F significantly reduced the incidence of SSI (RR = .401, 95% confidence interval (CI) = .190, .844; p = .016). Across four of six studies that reported SSI rates, there was no significant difference in SSI rates between ciNPT-MLAvs SOC (RR = .580, 95% CI = .222, 1.513; p = .265). SSC rates were evaluated in eight of 12 ciNPT-F studies that reported SSC rates. This meta-analysis of the eight ciNPT-F studies showed significantly reduced SSC rates with ciNPT-F vs SOC (RR = .332, 95% CI = .236, .467; p < 0.001). For ciNPT-MLA, five of six studies reported SSC rates. In those, there was no significant difference in SSC rates between ciNPT-MLA vs SOC (RR = .798, 95% CI = .458, 1.398; p = .425). These meta-analyses results showed a significant reduction in SSI and SSC rates in the ciNPT-F group vs SOC and no difference in SSI and SSC rates in the ciNPT-MLA group vs SOC. The reasons for these observed differences were not evaluated as part of this study. Future controlled clinical studies comparing outcomes between different ciNPT systems over closed orthopedic incisions would help to validate these study results.

## Introduction and background

Surgical site infections (SSIs) or other complications following total joint arthroplasty can result in significant morbidity for the patient and an enormous burden on the healthcare system. One study estimated that on average, SSI cases following primary knee replacement surgery cost eight times more than noninfected controls during the two-year follow-up period [1]. Standard regimens that include preoperative patient optimization, skin preparation with alcohol-based solutions, perioperative antibiotics, and minimizing wound drainage, have all been successful in reducing infection rates after joint arthroplasty. However, despite these efforts, postoperative complications are a persistent threat to the millions of people undergoing surgical interventions due to factors contributing to potential challenges in maintaining incision closure. Large symptomatic postoperative fluid collections frequently occur post-primary and revision total joint arthroplasty, providing an arena for bacterial growth and periprosthetic joint infection. Also, growing patient expectations coupled with advancements in technology and techniques are allowing for surgical procedures on patients with increasingly higher baseline surgical site complication (SSC) risk [2]. This is in addition to a rising prevalence of osteoarthritis in an aging population with increased longevity, increasing obesity rates, and an evolving profile of antibiotic-resistant bacteria.

Appropriate surgical wound and incision management during postoperative care are important to prevent complications, including SSI and wound dehiscence. Particularly for patients at higher risk of postoperative wound complications, wound dressings can play a critical role in closed incisions. The use of closed incision negative pressure therapy (ciNPT) to postoperatively manage the closed surgical incision has been expanding, as evidenced by a growing body of literature describing its use in numerous fields of surgery. Applying ciNPT over closed incisions acts as a barrier against external contamination while removing excess fluid that may contain infectious materials. Depending on construction, it can decrease lateral tension and mechanically offload the incision by approximating the wound edges [3]. A reduced incidence of seroma and superficial SSI in patients at high risk for postoperative complications has been reported in several studies following the use of ciNPT over hip or knee arthroplasty incisions [4-6].

However, the results and reported endpoints, as well as the type of ciNPT system used, are mixed across published orthopedic studies evaluating ciNPT [7-9]. There are primarily two different commercially available ciNPT systems that have been evaluated in the literature: ciNPT with foam dressing (ciNPT-F) and ciNPT with multi-layer gauze-based absorbent dressing (ciNPT-MLA). Both systems consist of a single-use, battery-powered device, but key product characteristic differences exist between each system, including dressing materials, level of negative pressure delivered, and method of exudate collection. Foam-based ciNPT has been used extensively at our facility with good results [10-12]. However, the device cost of ciNPT-F is greater than ciNPT-MLA, and there is ongoing pressure to provide stronger evidence supporting product selection decisions. Based on positive experiences with ciNPT-F [10,11] and significantly higher reported rates of incisional closure with foam-based versus non-foam-based ciNPT in a tissue model [13], we hypothesized that ciNPT-F versus ciNPT-MLA would demonstrate lower SSI and SSC rates compared to controls. The purpose of this systematic review and meta-analysis was to compare SSI and SSC rates after the use of ciNPT-F and ciNPT-MLA versus conventional dressings following hip and knee arthroplasty.

## Review

Literature searches

Two systematic literature searches were conducted to identify published studies that compared the effect of ciNPT vs standard of care (SOC) over closed incisions after hip or knee arthroplasty (KA). Public/Publisher MEDLINE (PubMed), Excerpta Medica dataBASE (EMBASE), and internal library QUOSA database were searched to identify relevant ciNPT-F studies, and EMBASE and Medical Literature Analysis and Retrieval System Online (MEDLINE) databases were used to identify relevant ciNPT-MLA studies published between January 2005 and July 2021. The QUOSA database was not used for the ciNPT-MLA literature search because it is an internal database; MEDLINE and EMBASE were selected to cover the best global ciNPT-MLA evidence.

Inclusion criteria consisted of published abstracts or manuscripts written in English that compared the effects of one of two manufacturer’s ciNPT systems (3M™ Prevena™ Incision Management System (3M, St. Paul, MN) or PICO^◊^ Single Use Negative Pressure Wound Therapy System (Smith & Nephew, Andover, MA)) over closed incisions versus SOC following primary or revision knee or HA. Exclusion criteria included meta-analysis, preclinical studies, veterinary studies, pediatric patient population, non-comparative studies, or the use of non-ciNPT devices. There were no restrictions on the inclusion or exclusion criteria with respect to patient or surgical risk factors for complications or follow-up time. Studies were excluded if the ciNPT treatment arm included a mixture of ciNPT types/brands or if SSI/SSC rates were not reported by type/brand, as were studies that described the use of ciNPT with products other than the two devices.

Titles and abstracts of publications identified in the databases were logged. After removing all duplicates, titles and abstracts of each paper were read to assess eligibility. The remaining publications were read to ensure all inclusion criteria and no exclusion criteria were met. References from identified publications were also reviewed for relevant studies that fit the inclusion criteria.

Data collection

One reviewer completed data extraction from all eligible studies and a second reviewer validated the findings. Disagreements were resolved by discussion between the two reviewers or by the addition of a third reviewer. The following data were extracted from each study: funding source, bias assessments, study design, publication status, study date range, number of study sites, study location, surgical procedures, high-risk enrollment criteria (if applicable), study objectives, control type, number of treatment days, follow-up period, number of patients/incisions, number of patients/incisions analyzed, and definition of SSC. Patient outcomes data such as number of SSCs, SSIs, dehiscence, seromas, hematomas, skin necrosis, readmissions, reoperations, deaths, amputations, type of SSI, and length of stay were extracted.

Risk of bias assessment

To assess the randomized controlled trial (RCT) risk of bias, the Cochrane Collaboration’s tool, designating “low”, “high”, or “unclear” risk, was used. Each RCT study included in the meta-analysis was assessed for selection bias based on a randomization process and allocation concealment. Performance bias results were based on a blinded assessment of outcomes and attrition bias was assessed by “lost to follow up” and incomplete datasets. Reporting bias was determined by comparing reported results to endpoints defined in the protocol. The impact of studies with a high risk of bias was assessed related to their overall impact on results using the “one-study-removed” procedure; no assumptions were made to fill in missing data. Data were used in the analyses as reported.

Statistical analysis

Primary grouping for analysis was based on the type of ciNPT device used. The endpoints evaluated were SSI rates and the reported composite SSC rates and were based on the availability of reported SSI and SSC endpoints. Further endpoint evaluations were not feasible due to the limited number of studies regarding ciNPT use post total knee or hip arthroplasty (HA).

To assess the effect of ciNPT versus SOC on dichotomous variables, weighted risk ratios (RR) were calculated to pool study and control groups in each publication for analysis. Treatment effects were combined, and a random effects model was used to assess the mean effect of (1) ciNPT-F versus SOC and (2) ciNPT-MLA versus SOC for all endpoints evaluated due to the assumed variability in study populations and procedures.

Random effects models were used regardless of the heterogeneity assessments. Forest plots of the risk ratios were generated with all studies. The I^2^ statistic was used to help assess heterogeneity. Fixed effects models were used for sensitivity analyses. Comprehensive Meta-Analysis Version 3.0 (Biostat Inc., Englewood, NJ) software was used to perform the analyses.

Search results

**Figure 1:**
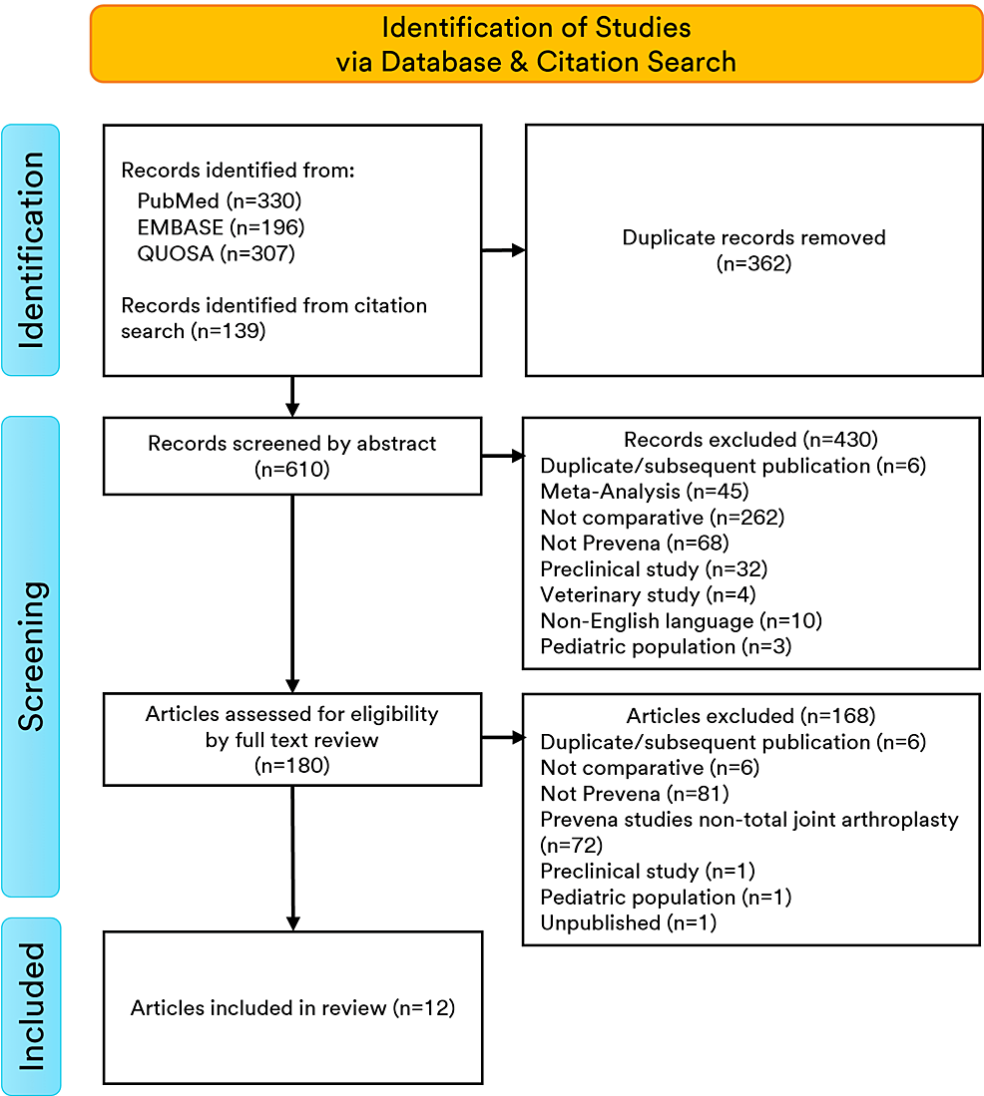
PRISMA flow diagram for ciNPT-F studies included in meta-analyses (n=12) PRISMA=Preferred Reporting Items for Systematic Reviews and Meta-Analyses, ciNPT-F=closed incision negative pressure therapy with foam dressing

The ciNPT-F-related search yielded 610 citations after duplicates were eliminated. After removing studies that did not meet inclusion criteria, 84 papers were identified for further evaluation. Of these, 12 were specific to ciNPT-F application over KA and/or HA incisions (Figure 1).

**Figure 2:**
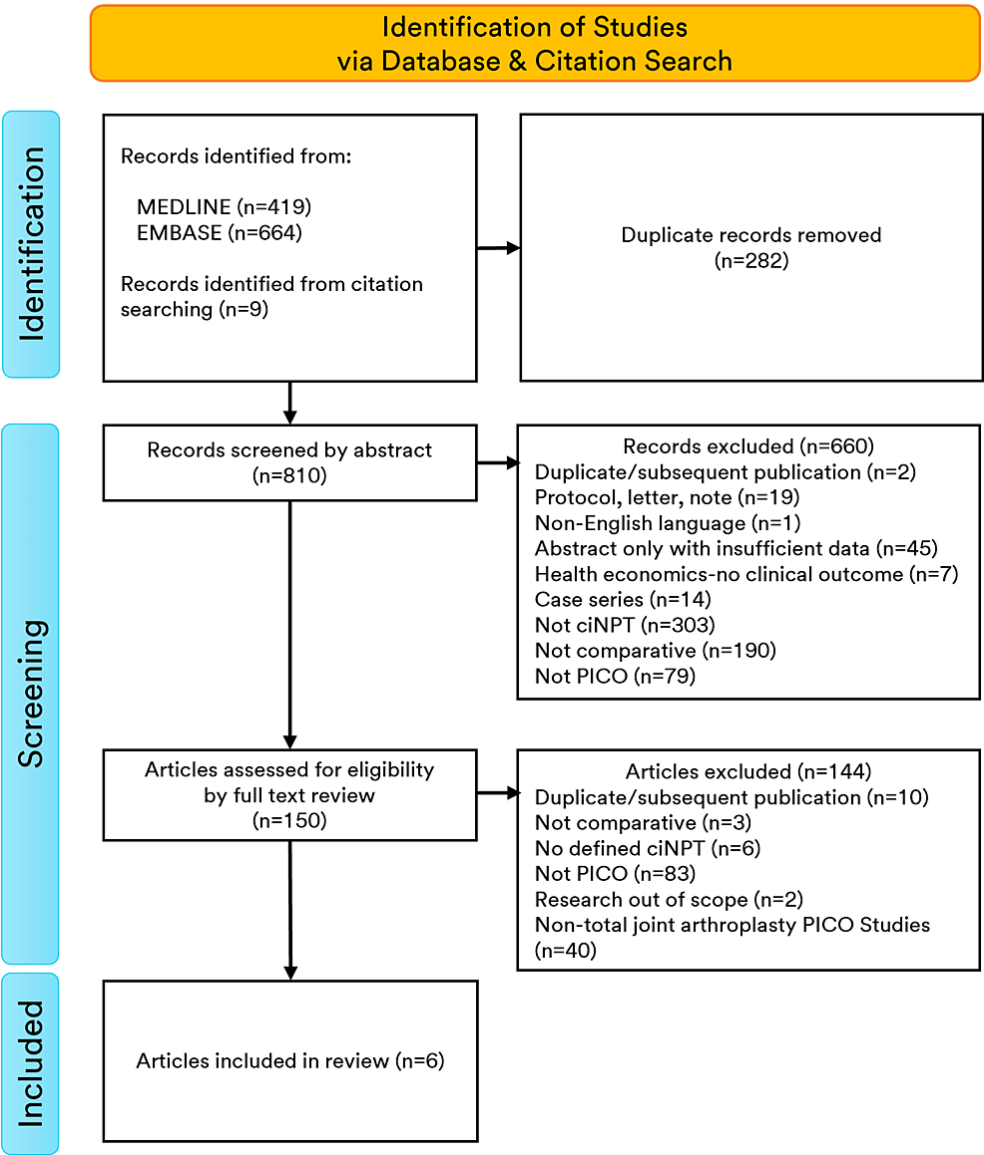
PRISMA flow diagram for ciNPT-MLA studies included in meta-analyses (n=6) PRISMA=Preferred Reporting Items for Systematic Reviews and Meta-Analyses, ciNPT-MLA=closed incision negative pressure therapy with multi-layer gauze-based absorbent dressing

The ciNPT-MLA-related search yielded 801 citations after duplicates were eliminated. After removing studies that did not meet the inclusion criteria for ciNPT-MLA comparative research 46 papers were identified for further evaluation. Application over KA and/or HA incisions (n=5) plus one added by reference search resulted in six ciNPT-MLA papers for analysis (Figure 2).

These 18 studies were included in the four meta-analyses. All studies with SSI and SSC incidence data were included in the related outcome assessments. Study characteristics and definitions of SOC for each of the studies are summarized in Table 1.

**Table 1:** Comparison of SSC and SSI reporting for (a) ciNPT-F vs SOC studies and (b) ciNPT-MLA vs SOC studies ant=anterior, AU=Australia, BR=Brazil, ciNPT-F=closed incision negative pressure therapy with foam dressing, ciNPT-MLA=closed incision negative pressure therapy with multi-layer gauze-based absorbent dressing, fem=femoral, GE=Germany, HA=hip arthroplasty, Hemiarth=hemiarthroplasty, hist=historical, IT=Italy, KA=knee arthroplasty, NR=not reported, post=posterior; Pro=prospective, RCT=randomized controlled trial, THA=total hip arthroplasty, TKA=total knee arthroplasty, Retro=retrospective, SSC=surgical site complication, SSI=surgical site infection, SOC=standard of care, UK=United Kingdom, UKA=unicompartmental knee arthroplasty, UniPat= unicompartmental knee arthroplasty with patellofemoral arthroplasty, US=United States

Study	Study Design	Country	Surgical Incision Type	ciNPT (n)	SOC (n)		SSI ciNPT (n (%))	SSI SOC (n (%))	SSC ciNPT (n (%))		SSC SOC (n (%))	SOC Dressing
ciNPT-F vs SOC Studies												
Anatone 2018 [14]	Retro	US	Primary TKA and THA	123	122		NR	NR	9 (7.3)		32 (26.2)	Hydrofiber dressing w/ silver
Cooper 2016 [10]	Retro	US	Revision TKA and THA	30	108		1 (3.3)	20 (18.5)	2 (6.7)		29 (26.9)	Hydrofiber dressing w/ silver
Curley 2018 [15]	Retro	US	TKA/UKA/UniPat	32	159		0 (0.0)	9 (5.7)	2 (6.3)		37 (23.3)	Dry sterile dressing
Doman 2021 [16]	Retro	US	Primary TKA	130	130		NR	NR	9 (6.9)		21 (16.2)	Hydrofiber wound dressing w/ silver
Higuera-Rueda 2021 [4]	RCT	US	Revision TKA	147	147		2 (1.4)	6 (4.1)	5 (3.4)		21 (14.3)	Silver-impregnated occlusive dressing
Manoharan 2016 [17]	Pro	AU	Primary TKA	21	36		NR	NR	1 (4.8)		1 (2.8)	Conventional dry Dressing
Newman 2017 [18]	RCT	US	Revision THA and TKA	79	80		0 (0.0)	1 (1.3)	8 (10.1)		19 (23.8)	Hydrofiber wound dressing w/ silver
Pachowsky 2012 [5]	RCT	GE	THA	9	10		NR	NR	NR		NR	Standard dry wound dressing
Pauser 2016 [19]	RCT	GE	Hemiarth. fem. neck fractures	11	10		NR	NR	NR		NR	Standard dressing - dry wound coverage
Redfern 2017 [6]	Pro w/ Hist Control	US	Primary THA and TKA	196	400		2 (1.0)	14 (3.5)	3 (1.5)		22 (5.5)	Traditional gauze dressing
Tyagi 2019 [20]	Retro	US	Primary direct ant approach THA	86	189		2 (2.3)	2 (1.1)	NR		NR	Hydrofiber wound dressing w/ silver
Tyagi 2020 [21]	Retro	US	Primary post approach THA	92	143		1 (1.1)	3 (2.1)	NR		NR	Hydrofiber wound dressing w/ silver
ciNPT-MLA vs SOC Studies												
Giannini 2018 [22]	RCT	IT	Revision hip and knee	50	50		NR	NR	NR		NR	Povidone-iodine gauze and patch wound dressing
Gillespie 2015 [23]	RCT	AU	Primary THA	35	35		2 (5.7)	3 (8.6)	24(68.6)		15 (42.9)	Hydrocolloid reinforced w/ 2-layer absorbent dressing, then w/ nonwoven dressing retention tape
Helito 2020 [24]	Pro w/ Hist Control	BR	Primary TKA	97	199		0 (0.0)	7 (3.5)	28(28.9)		91 (45.7)	Conventional dressings
Hester 2015 [25]	Retro	UK	Revision KA and HA	18	18		NR	NR	1 (5.6)		3 (16.7)	Blue gauze cotton wool and crepe bandaging for knees or pressure dressings for hips
Karlakki 2016 [26]	RCT	UK	Primary TKA and THA	102	107		0 (0.0)	5 (4.7)	2 (2.0)		9 (8.4)	Absorbent, self-adhesive dressing, or transparent film dressing
Keeney 2019 [27]	RCT	US	Primary/revision TKA/THA	185	213		7 (3.8)	8 (3.8)	22(11.9)		27 (12.7)	Nonadherent incisional cover, gauze, and absorbable dressing
Total SSI/SSC ciNPT-F (n, (%))							8/662 (1.2)	55/1226 (4.5)	39/758 (5.1)		182/1182 (15.4)	
Total SSI/SSC ciNPT-MLA (n, (%))							9/419 (2.1)	23/554 (4.2)	77/437 (17.6)		145/572 (25.3)	

Four of the ciNPT-F studies were RCTs, two were prospective studies, and six were retrospective studies. Of the ciNPT-MLA studies, four were RCTs, one was prospective, and one was retrospective. The ciNPT-F vs SOC studies comprised 956 patients in the ciNPT-F cohort and 1,534 in the SOC cohort, and the ciNPT-MLA vs SOC studies comprised 487 in the ciNPT-MLA cohort and 622 in the SOC cohort. ciNPT-F studies took place primarily in the US (n=9), plus Germany (n=2), and Australia (n=1). Only one of the ciNPT-MLA studies took place in the US with the rest being from Italy (n=1), Australia (n=1), Brazil (n=1), and the UK (n=2).

Arthroplasty procedures varied across the ciNPT-F studies and comprised primary TKA and/or THA, revision TKA and/or THA, unicompartmental knee arthroplasty (UKA), patellofemoral arthroplasty (UniPat), and hemiarthroplasty for femoral neck fractures. The ciNPT-MLA studies comprised revision and primary hip and KA patients.

Outcomes

In the studies evaluating SSC, investigators reported overall SSC rates where SSC was defined as a composite endpoint that included multiple types of complications. The meaning of SSC was defined individually in each study. Types of SSCs included the following: SSI, suture granuloma, focal swelling/bullae, suture reaction/stitch abscess, skin necrosis, blistering, non-healing wound, post-operative antibiotics, edema/swelling, hyperemia, periprosthetic joint infection, seroma, hematoma, dehiscence, cellulitis, prolonged drainage and return to the operating room.

Meta-analyses results are listed in Table 2.

**Table 2:** Meta-analyses results of random effects models ciNPT-F=closed incision negative pressure therapy with foam dressing, ciNPT-MLA=closed incision negative pressure therapy with multi-layer gauze-based absorbent dressing, SSC=surgical site complication; SSI=surgical site infection

Endpoint	Product	Number of Studies	Risk Ratio	Lower Limit	Upper Limit	I^2^	p-value	Relative Risk Reduction	Lower Limit	Upper Limit
SSI	ciNPT-F	7	0.401	0.190	0.844	0.000	0.016	60%	16%	81%
	ciNPT-MLA	4	0.580	0.222	1.513	16.725	0.265	42%	-51%	78%
SSC	ciNPT-F	8	0.332	0.236	0.467	0.000	<0.001	67%	53%	76%
	ciNPT-MLA	5	0.798	0.458	1.390	72.013	0.425	20%	-39%	54%

**Figure 3:**
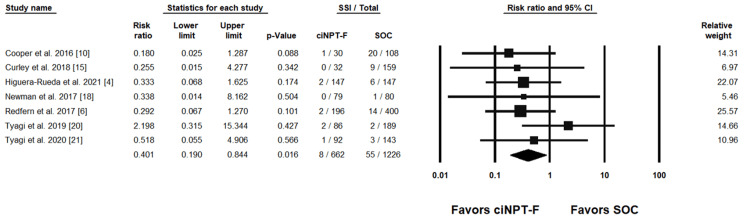
ciNPT-F v SOC surgical site infection results [4,6,10,15,18,20,21] ciNPT-F=closed incision negative pressure therapy with foam dressing, SSI=surgical site infection, CI=confidence interval, SOC=standard of care

**Figure 4:**
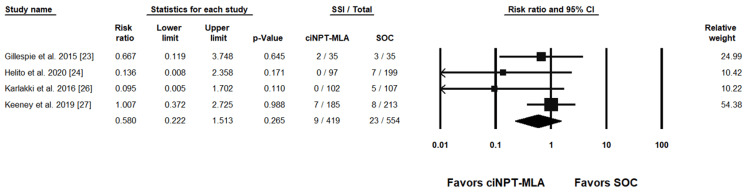
ciNPT-MLA vs SOC surgical site infection results [23,24,26,27] ciNPT-MLA=closed incision negative pressure therapy with multi-layer absorbent dressing, SSI=surgical site infection, CI=confidence interval, SOC=standard of care

**Figure 5:**
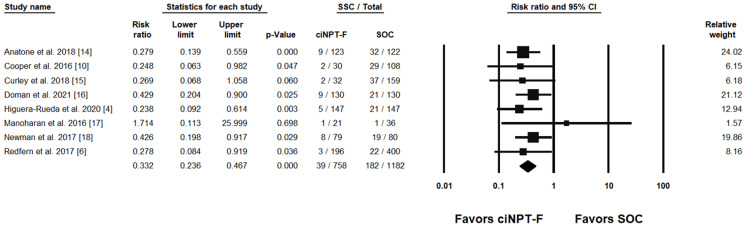
ciNPT-F vs SOC surgical site complication results [4,6,10,14-18] ciNPT-F=closed incision negative pressure therapy with foam dressing, SSC=surgical site complication, CI=confidence interval, SOC=standard of care

**Figure 6:**
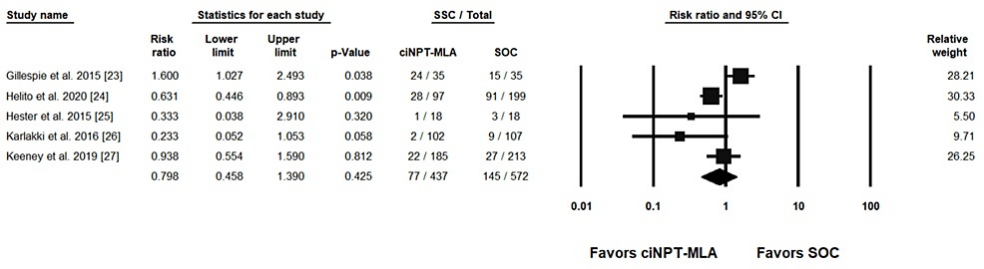
ciNPT-MLA vs SOC surgical site complication results [23-27] ciNPT-MLA=closed incision negative pressure therapy with multi-layer absorbent dressing, SSC=surgical site complication, CI=confidence interval, SOC=standard of care

Individual study results with the combined summary result are presented in forest plots for SSI rates in Figure 3 and Figure 4 and for SSC rates in Figure 5 and Figure 6.

Seven ciNPT-F studies reported SSI rates and were included in the meta-analysis. The weighted RR was 0.401 (95% confidence interval (CI) = .190, .844; p = .016) indicating a significant difference in SSI rates in favor of ciNPT-F. Four ciNPT-MLA studies reported SSI rates and were included in the meta-analysis. The weighted RR was 0.580 (95% CI = .222, 1.513; p = .265) indicating no difference between ciNPT-MLA and SOC with respect to SSI rates.

Eight of 12 ciNPT-F studies reported total SSC rates, and across the eight studies, the weighted RR was 0.332 (95% CI = .236, .467; p < 0.001). This indicates a significant difference in favor of ciNPT-F versus SOC. Five of six ciNPT-MLA studies reported SSC rates and the weighted RR was 0.798 (95% CI = .458, 1.398; p = .425), indicating no significant difference in SSC rates between ciNPT-MLA and SOC. For all SSI and SSC rate analyses, the results from the fixed effects model were consistent with the results of the random effects models.

Of all studies analyzed for bias, only the Karlakki (2016) ciNPT-MLA RCT presented a high risk of bias. For all other types of bias analyzed, all other studies had a “low” or “unclear” risk of bias.

While there was low heterogeneity in the ciNPT-F studies that reported SSI or SSC rates (I2 = 0.0000 and 0.0000, respectively), there was higher heterogeneity across ciNPT-MLA studies, especially among those reporting SSC (I2 = 72.013). The heterogeneity could be due to the variation in study outcomes between the studies. All ciNPT-F studies reported a lower SSC rate versus SOC, with six of the eight studies reporting a significantly lower SSC rate. Hence, the low heterogeneity in ciNPT-F studies related to SSC outcomes.

Discussion

Preventing SSI and other complications remains a significant ongoing concern for orthopedic surgeons due to cost and the growing numbers and complexities of patients seeking a joint replacement. In developed countries, between 2009 and 2019, the average number of hip replacements increased by 22% and the number of knee replacements by 35% [28], and hip and KA utilization rates are expected to increase even further with the uptick in worldwide demand.

To date, this is the largest meta-analysis comparing clinical outcomes between two different ciNPT products in orthopedic surgery. The findings support our hypothesis of increased SSC and SSI reduction with ciNPT-F. The results also complement outcomes of a recently published meta-analysis by Elhage and colleagues (2022) [29] that demonstrated significantly fewer overall complications and lower incidence of persistent wound drainage for total hip or KA incisions managed with ciNPT-F, compared to ciNPT-MLA or conventional dressings. The Elhage study analyzed data from six RCTs whereas our current meta-analysis analyzed results from additional evidence, including the Higuera-Rueda (2021) RCT and prospective and retrospective studies.

Of the seven ciNPT-F studies in this meta-analysis, five studies (Higuera-Rueda (2021), Cooper (2016), Curley (2018), Tyagi (2019), and Tyagi (2020)) reported a follow-up time in line with the CDC-recommended 90-day surveillance period for determining infections as specified for hip and knee prostheses. Follow-up time was 60 days for Redfern (2017) and 84 days for Newman (2017). One of four of the ciNPT-MLA studies (Helito 2020) followed the CDC-recommended ≥ 90-day surveillance period to determine SSI and the reported follow-up period for the other three studies ranged from 35 to 42 days. The greater variation in postoperative follow-up times among the ciNPT-MLA studies is aligned with the finding of greater heterogeneity across ciNPT-MLA studies, weakening the data quality compared to the ciNPT-F studies, with respect to the SSI endpoint. Overall, the results from ciNPT-F studies suggested greater reliability due in part to more consistent adherence to a 90-day follow-up period.

Performance bias results were based on a blinded assessment of outcomes, which was unreported in all but the Karlakki (2016) study and therefore did not yield any strong conclusions. The high risk for reporting bias in the Karlakki (2016) study was a result of the number of analyzed surgeries (102 in the study group and 107 in the control group) being lower than the number of enrolled surgeries (n = 110) for each group. The Karlakki study was included in both SSI and SSC assessments. One-study-removed analysis indicated that this study did not change the overall results, but it did contribute to a positive trend for the ciNPT-MLA group when included.

The Higuera-Rueda (2021) RCT is the highest-quality study included in the ciNPT-F analysis. It is a well-designed multi-site ciNPT-F study that showed that the primary endpoint of investigator-assessed 90-day occurrence of SSC was significantly lower in the ciNPT-F group versus the control group (silver-impregnated antimicrobial dressing), demonstrating outcomes that are aligned with the results of this meta-analysis. Although a comparison of individual SSC types (SSI, superficial SSI, deep SSI, wound dehiscence, development of seroma or hematoma requiring drainage, skin necrosis, or continued wound drainage) between both cohorts in the Higuera-Rueda study did not show a statistically significant reduction within the ciNPT-F cohort, there was a positive trend for SSI reduction which contributed to overall lower rates of 90-day SSC in the meta-analysis.

The findings from this meta-analysis have reinforced our institution’s practice of using ciNPT-F to manage closed incisions in high-risk total joint arthroplasty patients, with the goal of reducing their risk of SSC and SSI. Our current approach is to use ciNPT-F in most revision arthroplasty patients and, as described by Anatone (2018), a minority of primary arthroplasty patients who carry multiple risk factors for SSC or SSI.

Still, several limitations may impact the results of this study, including the difference in population size between the ciNPT-F vs SOC group (n=2,490) and the ciNPT-MLA vs SOC group (n=1,109). In addition, the rate of revision total joint arthroplasty (TJA) is underrepresented in ciNPT-MLA studies compared to ciNPT-F studies. Different definitions of SSC and SSI across studies could also have affected the accuracy of the results. Also, the wide variance between the types of SSCs (i.e., blistering vs return to the operating room) as well as surgical procedure variances could limit the results. Results could be further limited by differences in study design, target populations, administrative methodology, and timing of outcome measurements. The available data were insufficient to conduct subgroup analysis of different types of SSCs, except SSIs. Clinical studies are recommended to determine the comparative outcomes between different ciNPT systems over closed incisions following TJA.

## Conclusions

We performed four meta-analyses to compare SSI and SSC rates with the use of ciNPT-F versus SOC or ciNPT-MLA versus SOC over closed incisions following HA and KA. The meta-analyses demonstrated a statistically significant reduction in the incidence of SSIs and SSCs in the ciNPT-F versus SOC group, but no significant difference in SSI or SSC rates in the ciNPT-MLA group compared to the SOC group.

This is the first set of meta-analyses comparing the outcomes of two different types of ciNPT systems versus SOC in managing closed incisions following HA and KA. Results suggest there could be differences in SSC rates between types of ciNPT used, but considerably more study is needed to validate these outcomes.
